# Association analysis of leaf aromatic substances in cultivated and weedy types of *Perilla* crop using SSR markers

**DOI:** 10.1016/j.heliyon.2024.e34995

**Published:** 2024-07-23

**Authors:** Jungeun Cho, Kyu Jin Sa, Hyeon Park, Tae Hyeon Heo, Sookyeong Lee, Ju Kyong Lee

**Affiliations:** a Department of Applied Plant Sciences, College of Agriculture and Life Sciences, Kangwon National University, Chuncheon 24341, South Korea; b Interdisciplinary Program in Smart Agriculture, Kangwon National University, Chuncheon 24341, South Korea; c Department of Crop Science, College of Ecology & Environmental Sciences, Kyungpook National University, Sangju 37224, South Korea; d National Agrobiodiversity Center, National Institute of Agricultural Sciences, RDA, Jeonju 54874, South Korea

**Keywords:** *Perilla* leaves, Volatile compounds, Genetic diversity, *Perilla* SSR marker

## Abstract

In East Asia, particularly South Korea, the two cultivated varieties of *Perilla* are commonly grown. They are clearly distinguished by their aromatic substances and have different uses as leafy vegetables or oil crop. This study was performed for the development of simple sequence repeat (SSR) markers linked to volatile compounds in *Perilla* leaves that show differences between cultivated var. *frutescens* (CF), weedy var. *frutescens* (WF), and weedy var. *crispa* (WC) of *Perilla*. Fifty *Perilla* SSR primer sets were used to analyze genetic diversity for the 80 *Perilla* accessions of the three types. A total of 276 alleles were detected, with an average of 5.5 alleles per locus. The average genetic diversity values for CF, WF, and WC accessions were 0.402, 0.583, and 0.437, respectively. WF accessions exhibited the highest genetic diversity among the three types of the *Perilla* crop. Phylogenetic tree analysis classified 80 *Perilla* accessions of the three types into four groups, showing 37.2 % genetic similarity. Three types of the *Perilla* crop were clearly distinguished except for outstanding accessions. Through the application of an association analysis involving 50 *Perilla* SSR primer sets and five volatile compounds (perilla aldehyde, perilla ketone, myristicin, dill apiol, (Z,E)-α-farnesene) in the three types of the *Perilla* accessions, we detected 11 significant marker-trait associations duplicated in both Q GLM and Q + K MLM methods. These findings serve as valuable insights for identifying the aromatic substances in *Perilla* plants originating from various regions of South Korea.

## 1 Introduction

*Perilla* crop (*Perilla frutescens* L.) is a self-pollinating annual plant divided into two cultivated varieties: cultivated var. *frutescens* (CF) and cultivated var. *crispa* (CC). These varieties are distinguished by their morphology and utilization in East Asia [[1], [2], [3], [4]]. For centuries, *Perilla* has been valued in East Asia for food and industrial purposes. CF cultivation is predominantly in South Korea, where CF is used extensively with its seeds employed for producing perilla seed oil and its leaves consumed as a vegetable. Conversely, CC is primarily cultivated in Japan and is most popular for use as a fresh leafy vegetable or as a leafy vegetable for pickling [2,4]. In South Korea, CF is highly regarded for its aromatic leaves, while CC leaves are less favored. In contrast, Japanese people appreciate the scent of CC leaves but dislike that of CF leaves [1,4,5]. Consequently, CC is neither cultivated nor utilized in South Korea, while CF is not cultivated or utilized in Japan [[2], [3], [4]]. Additionally, while the wild *Perilla* species remains unidentified, two weedy types, weedy var. *frutescens* (WF) and weedy var. *crispa* (WC), are commonly found in East Asia [2,[6], [7], [8]].

In many plant species, essential oils embody the art of nature, producing enticing aromas that are derived from a complex blend of secondary metabolites that are synthesized and released by aromatic and spice plants [5,9]. In *Perilla* crop, these aromatic compounds (or volatile compounds) are rich and resplendent and produce the unique aromas and flavors that captivate the olfactory senses of Koreans and Japanese [1,5,10]. *Perilla* essential oil has been reported to contain high levels of antioxidants, anti-cancer, anti-inflammatory, insecticidal, and antibacterial agents [[11], [12], [13]]. The pharmacological properties of *Perilla* leaves derive from the unique combination and ratio of volatile compounds. Notably volatile compounds include perilla ketone (PK), perilla aldehyde (PA), benzaldehyde, limonene, and β-caryophyllene [5,[14], [15], [16]]. Moreover, depending on the variations in the content of these compounds, the two cultivated varieties (CF and CC) of *Perilla* have been classified into seven chemotypes, each distinguished by the presence of specific volatile compounds in their leaves. These compounds include PK, PA, perillene, piperitenone, elsholtzia ketone, citral, and phenylpropanoid [5,17,18].

Selecting useful genetic resources is crucial for efficient cultivar development. Recently, polymerase chain reaction (PCR) based molecular markers have played a very important role in selective breeding programs for many crops. In *Perilla* crop, many microsatellite or simple sequence repeat (SSR) primer sets have recently been developed [[19], [20], [21], [22]]. They are being used to develop molecular markers related to useful traits such as leaf and seed characteristics and fatty acid content of the three types (CF, WF, WC) of the *Perilla* [[23], [24], [25], [26]]. In *Perilla* crop, in particular, *Perilla* SSR markers exhibit characteristics of codominance, high repetitiveness, and high DNA polymorphism [[19], [20], [21], [22]]. Also they can be used with PCR technology, and so they are useful in genetic investigations, including aspects such as genetic diversity (GD), polymorphism information content (PIC), phylogenetic research, population structure analysis (PSA), and association mapping analysis (AMA) for cultivated and weedy types of *Perilla* [[23], [24], [25], [26], [27]].

In South Korea, CF has attracted considerable interest as a versatile crop for production of both edible oil and fresh vegetables. Establishing *Perilla* SSR markers associated with these beneficial traits will be essential for the development of new *Perilla* cultivars for leafy vegetables rich in health-enhancing volatile compounds. Hence, in our research, we employed *Perilla* SSR primer sets to identify molecular markers associated with aromatic substances (or volatile compounds) in *Perilla* leaves and revealed differences between and within accessions of the three types (CF, WF, WC) of the *Perilla* crop, which originates from diverse regions across South Korea.

## 2 Materials and methods

### 2.1 Plant materials and Perilla DNA extraction

Our study utilized a total of 80 *Perilla* accessions of the three types used in a previous study of Sa et al. [5]. These *Perilla* accessions were classified into three types: 40 CF accessions, 20 WF accessions, and 20 WC accessions. Comprehensive details regarding these *Perilla* accessions are available in Supplementary data, Table S1. The 80 *Perilla* accessions of the three types (CF, WF, WC) used in this study were collected in various regions of South Korea from 2016 to 2021 and safely stored in the RDA-Genebank (http://genebank.rda.go.kr), Jeonju, Republic of Korea for long term seed preservation. To extract genomic DNA from *Perilla* accessions, young leaf tissue was used using the Plant DNAzol Reagent protocols (GibcoBRL Inc., Grand Island, NY, USA) as described by Lee and Ohnishi [7], with slight modifications.

### 2.2 SSR amplification and DNA electrophoresis

The Supplementary data, Table S2, provides a comprehensive list of 50 *Perilla* SSR loci employed in this study. Among them, seven *Perilla* SSR primer sets (KNUPE162, KNUPE163, KNUPE164, KNUPE165, KNUPE166, KNUPE167, KNUPE168) were used for analysis for the first time in the current study. PCR amplification of the *Perilla* SSR primer sets was conducted using the EX Taq PCR kit (Takara Co, Japan), with a 20 μL reaction mixture being prepared for each plant DNA sample in order to perform the PCR amplification. The mixture included 20 ng of *Perilla* genomic DNA, 10 × EX Taq buffer, 0.2 mM of the dNTP mix, 0.5 μM of each forward and reverse primer, and 1 unit of EX Taq polymerase. The PCR procedure started with an initial denaturation at 94 °C for 5 min, followed by cycles of denaturation at 94 °C for 1 min, annealing at 65 °C for 1 min, and extension at 72 °C for 2 min. During the annealing stage, the temperature was gradually reduced by 1 °C after each cycle until it reached a final annealing temperature of 55 °C. This process was repeated for a total of 36 cycles. After completing the PCR cycles, a final extension was conducted at 72 °C for 5 min. Subsequently, DNA electrophoresis was conducted using a mini vertical electrophoresis system (MGV-202-33, CBS Scientific Company, San Diego, USA). To prepare for electrophoresis, a solution was made by mixing 3 μL PCR product with 3 μL of formamide loading dye. The loading dye contained 0.02 % bromophenol blue (BPB), 0.02 % xylene C, 98 % formamide, and 5 mM NaOH. Subsequently, 3 μL of the prepared PCR sample was loaded onto a 6 % acrylamide-bisacrylamide gel (19:1) in 0.5 X Tris-borate-EDTA (TBE) buffer, and electrophoresis was carried out at 250 V for 40–60 min. The resulting isolated DNA fragments were stained with ethidium bromide (EtBr) for visualization.

### 2.3 Statistical analysis

DNA fragments produced by the 50 *Perilla* SSR primer sets were assessed as being either present (1) or absent (0). The GD for each type of accession was subsequently calculated using a formula that was developed by Nei [28]:

Genetic diversity (GD) = 1－∑ *P*_*i*_^2^,

where *Pi* represents the frequency of the ith SSR allele in a group of accessions. The number of alleles, major allele frequency (MAF), and PIC were computed for the 80 *Perilla* accessions of the three types (CF, WF, WC) and the 50 *Perilla* SSR loci using Power Marker version 3.25 [29]. The genetic similarities (GS) between each pair of *Perilla* accessions were determined using the Dice similarity index [30]. This similarity matrix that was then utilized to construct a dendrogram based on the unweighted pair group method with arithmetic averages (UPGMA), using the SAHN-Clustering tool in NTSYS-pc version 2.1 [31]. Moreover, for the purpose of investigating the clustering pattern of the *Perilla* accessions of the three types (CF, WF, WC), we employed the POPGENE 1.32 [32] to construct a phylogenetic tree, which is based on a genetic distance matrix proposed by Nei [28]. Additionally, MEGA-X v11.0.11 [33] was utilized to visualize the generated phylogenetic tree. Additionally, PSA was performed on the 80 *Perilla* accessions of the three types (CF, WF, WC) using the STRUCTURE v2.3 software [34]. The optimal K value for the entire population was identified using the delta K (ΔK) simulation method [35] with the STRUCTURE HARVESTER web-based software program (https://taylor0.biology.ucla.edu/structureHarvester/). Lastly, marker-trait associations (MTAs) were assessed using TASSEL 3.0 software [36] through both the general linear model (Q GLM) and the mixed linear model (Q + K MLM). The methodologies for Q GLM and Q + K MLM applied in this study were based on the approach outlined by Jang et al. [26].

## 3 Results

### 3.1 Genetic diversity of the three types of the Perilla using Perilla SSR markers

In our research, we employed 50 *Perilla* SSR loci to evaluate the GD index, which included parameters such as the number of alleles, MAF, GD, and PIC for 80 *Perilla* accessions of the three types (CF, WF, WC) from South Korea (Supplementary data, Table S1). The 50 *Perilla* SSR loci identified a total of 276 alleles among the 80 *Perilla* accessions. The number of alleles per *Perilla* SSR locus varied from three (KNUPF19, KNUPF20, KNUPF54, KNUPF60, KNUPF127, KNUPF136, KNUPF163, KNUPF164, KWPE56, GBPFM134) to 12 (KNUPF33), with an average of 5.5 alleles per locus. The DNA band sizes exhibited a range from 95 to 320 base pairs. The GD values for each *Perilla* SSR locus spanned from 0.165 (KNUPF125) to 0.830 (KNUPF2), with an average GD value of 0.596. The PIC values for each *Perilla* SSR locus exhibited a range from 0.162 (KNUPF125) to 0.811 (KNUPF2), with a mean value of 0.550. The MAF values for each *Perilla* SSR locus spanned from 0.288 (KWPE57) to 0.913 (KNUPF125), with an average MAF of 0.546 (Table 1).

**Table 1:** Characteristics of 50 *Perilla* SSR primer sets including allele size range, allele number, GD, PIC and MAF among 80 accessions of the three types of the *Perilla* collected from South Korea.

SSR loci	Allele size (bp)	Allele No	GD	PIC	MAF
KNUPF2	120–170	10	0.830	0.811	0.300
KNUPF3	170–200	9	0.826	0.805	0.300
KNUPF4	160–190	8	0.787	0.757	0.325
KNUPF5	170–190	6	0.771	0.737	0.325
KNUPF11	250–290	5	0.562	0.523	0.625
KNUPF14	170–190	5	0.631	0.595	0.563
KNUPF16	120–180	6	0.639	0.581	0.500
KNUPF18	190–280	5	0.432	0.386	0.725
KNUPF19	170–190	3	0.449	0.404	0.713
KNUPF20	180–200	3	0.318	0.290	0.813
KNUPF23	180–220	6	0.705	0.675	0.488
KNUPF26	140–170	5	0.346	0.328	0.800
KNUPF29	190–320	5	0.685	0.640	0.475
KNUPF30	180–280	5	0.650	0.596	0.500
KNUPF31	140–170	8	0.619	0.577	0.563
KNUPF32	190–210	5	0.531	0.491	0.650
KNUPF33	130–220	12	0.809	0.785	0.300
KNUPF37	220–320	6	0.618	0.560	0.538
KNUPF39	180–260	10	0.741	0.709	0.425
KNUPF42	140–180	6	0.641	0.613	0.563
KNUPF53	180–210	8	0.738	0.698	0.400
KNUPF54	120–190	3	0.391	0.338	0.750
KNUPF56	220–300	5	0.366	0.349	0.788
KNUPF60	170–270	3	0.593	0.506	0.463
KNUPF62	210–300	4	0.678	0.618	0.425
KNUPF72	220–300	4	0.573	0.496	0.550
KNUPF82	140–180	8	0.757	0.731	0.425
KNUPF85	150–210	6	0.581	0.537	0.600
KNUPF125	190–220	5	0.165	0.162	0.913
KNUPF127	140–280	3	0.545	0.474	0.600
KNUPF128	180–250	4	0.569	0.475	0.475
KNUPF130	130–190	5	0.590	0.535	0.575
KNUPF131	190–270	5	0.643	0.573	0.425
KNUPF133	160–180	7	0.649	0.585	0.438
KNUPF136	180–210	3	0.261	0.238	0.850
KNUPF143	210–270	6	0.698	0.660	0.475
KNUPF146	190–320	6	0.633	0.600	0.563
KNUPF162	190–300	5	0.581	0.537	0.600
KNUPF163	180–220	3	0.603	0.536	0.538
KNUPF164	160–170	3	0.503	0.451	0.663
KNUPF165	110–180	5	0.554	0.500	0.613
KNUPF166	210–260	4	0.573	0.515	0.588
KNUPF167	190–270	5	0.685	0.632	0.438
KNUPF168	140–210	4	0.673	0.613	0.413
KWPE56	95–140	3	0.466	0.395	0.675
KWPE57	140–180	9	0.815	0.792	0.288
GBPFM111	160–200	6	0.494	0.462	0.688
GBPFM134	170–190	3	0.527	0.453	0.613
GBPFM179	170–250	8	0.780	0.747	0.313
GBPFM201	150–200	5	0.506	0.445	0.650
Average		5.5	0.596	0.550	0.546

GD: Genetic diversity, PIC: Polymorphism Information Content, MAF: Major allele frequency.

Furthermore, we analyzed the GD among the three types (40 CF samples, 20 WF samples, and 20 WC samples) using the 50 *Perilla* SSR primer sets to identify genetic variation by considering parameters such as allele number, GD, PIC, and MAF for the 80 Korean *Perilla* accessions (Table 2). In our analysis of the result, we observed that the average allele numbers for the CF, WF, and WC accessions were 3.8, 4.2, and 3.7, respectively. The average GD values were calculated at 0.402, 0.583, and 0.437 for the respective CF, WF, and WC accessions. The average PIC values were found to be 0.368, 0.531, and 0.403 for the respective CF, WF, and WC accessions. The average MAF values were determined to be 0.710, 0.539, and 0.690 for the respective CF, WF, and WC accessions (Table 2).

**Table 2:** Genetic variation obtained from each SSR primer set among 80 accessions of three types (40 CF samples, 20 WF samples, and 20 WC samples) of the *Perilla* crop collected from South Korea.

SSR Loci	Cultivated var. *frutescens* (n = 40)				Weedy var. *frutescens* (n = 20)				Weedy var. *crispa* (n = 20)			
	Allele No	GD	PIC	MAF	Allele No	GD	PIC	MAF	Allele No	GD	PIC	MAF
KNUPF2	7	0.755	0.724	0.400	8	0.800	0.777	0.350	7	0.780	0.748	0.300
KNUPF3	7	0.669	0.636	0.525	7	0.820	0.797	0.300	7	0.760	0.726	0.350
KNUPF4	6	0.711	0.666	0.425	5	0.640	0.603	0.550	3	0.405	0.368	0.750
KNUPF5	4	0.666	0.607	0.450	5	0.755	0.716	0.350	5	0.775	0.739	0.300
KNUPF11	3	0.096	0.094	0.950	5	0.590	0.553	0.600	4	0.475	0.440	0.700
KNUPF14	2	0.095	0.090	0.950	4	0.695	0.633	0.350	5	0.595	0.561	0.600
KNUPF16	6	0.701	0.657	0.450	3	0.515	0.424	0.600	2	0.095	0.090	0.950
KNUPF18	3	0.359	0.310	0.775	5	0.645	0.590	0.500	3	0.265	0.247	0.850
KNUPF19	2	0.095	0.090	0.950	2	0.455	0.352	0.650	3	0.645	0.572	0.450
KNUPF20	3	0.224	0.207	0.875	3	0.485	0.406	0.650	3	0.265	0.247	0.850
KNUPF23	4	0.498	0.454	0.675	5	0.715	0.668	0.400	4	0.575	0.526	0.600
KNUPF26	4	0.229	0.220	0.875	4	0.525	0.480	0.650	2	0.320	0.269	0.800
KNUPF29	5	0.595	0.522	0.525	4	0.565	0.509	0.600	2	0.375	0.305	0.750
KNUPF30	2	0.219	0.195	0.875	5	0.765	0.725	0.300	4	0.345	0.326	0.800
KNUPF31	2	0.049	0.048	0.975	7	0.730	0.691	0.400	5	0.675	0.634	0.500
KNUPF32	2	0.219	0.195	0.875	3	0.445	0.381	0.700	5	0.595	0.561	0.600
KNUPF33	8	0.748	0.714	0.400	5	0.540	0.508	0.650	6	0.755	0.718	0.350
KNUPF37	3	0.184	0.174	0.900	4	0.615	0.544	0.500	3	0.185	0.177	0.900
KNUPF39	9	0.800	0.773	0.275	6	0.720	0.676	0.400	3	0.265	0.247	0.850
KNUPF42	2	0.139	0.129	0.925	4	0.705	0.650	0.350	6	0.745	0.709	0.400
KNUPF53	6	0.545	0.517	0.650	7	0.795	0.766	0.300	4	0.575	0.526	0.600
KNUPF54	3	0.526	0.431	0.575	3	0.265	0.247	0.850	1	0.000	0.000	1.000
KNUPF56	4	0.306	0.288	0.825	4	0.610	0.554	0.550	2	0.095	0.090	0.950
KNUPF60	2	0.289	0.247	0.825	3	0.460	0.410	0.700	1	0.000	0.000	1.000
KNUPF62	3	0.395	0.347	0.750	4	0.660	0.610	0.500	3	0.340	0.314	0.800
KNUPF72	4	0.341	0.317	0.800	3	0.585	0.495	0.450	3	0.335	0.303	0.800
KNUPF82	8	0.829	0.806	0.250	5	0.755	0.713	0.300	3	0.185	0.177	0.900
KNUPF85	4	0.143	0.139	0.925	4	0.685	0.632	0.450	5	0.545	0.517	0.650
KNUPF125	3	0.096	0.094	0.950	3	0.185	0.177	0.900	3	0.265	0.247	0.850
KNUPF127	2	0.255	0.222	0.850	3	0.565	0.482	0.550	3	0.335	0.303	0.800
KNUPF128	4	0.456	0.403	0.700	3	0.515	0.424	0.600	3	0.265	0.247	0.850
KNUPF130	3	0.096	0.094	0.950	4	0.715	0.661	0.350	4	0.465	0.420	0.700
KNUPF131	3	0.615	0.534	0.450	4	0.535	0.498	0.650	4	0.535	0.498	0.650
KNUPF133	3	0.421	0.365	0.725	6	0.700	0.651	0.400	3	0.185	0.177	0.900
KNUPF136	1	0.000	0.000	1.000	3	0.485	0.406	0.650	3	0.395	0.347	0.750
KNUPF143	4	0.496	0.452	0.675	5	0.665	0.619	0.500	5	0.540	0.508	0.650
KNUPF146	3	0.296	0.265	0.825	5	0.630	0.587	0.550	5	0.665	0.619	0.500
KNUPF162	2	0.180	0.164	0.900	3	0.535	0.436	0.550	5	0.660	0.601	0.450
KNUPF163	2	0.439	0.342	0.675	3	0.405	0.368	0.750	3	0.505	0.442	0.650
KNUPF164	2	0.180	0.164	0.900	3	0.515	0.424	0.600	3	0.605	0.527	0.500
KNUPF165	4	0.624	0.564	0.525	4	0.410	0.379	0.750	3	0.485	0.406	0.650
KNUPF166	3	0.141	0.136	0.925	4	0.615	0.544	0.500	4	0.610	0.554	0.550
KNUPF167	5	0.619	0.584	0.575	4	0.580	0.535	0.600	2	0.180	0.164	0.900
KNUPF168	4	0.671	0.619	0.475	3	0.585	0.495	0.450	4	0.475	0.440	0.700
KWPE56	3	0.551	0.461	0.550	3	0.445	0.381	0.700	2	0.180	0.164	0.900
KWPE57	6	0.746	0.711	0.400	6	0.820	0.795	0.250	5	0.665	0.619	0.500
GBPFM111	3	0.304	0.282	0.825	3	0.185	0.177	0.900	4	0.525	0.480	0.650
GBPFM134	3	0.374	0.343	0.775	3	0.265	0.247	0.850	3	0.265	0.247	0.850
GBPFM179	6	0.689	0.647	0.475	4	0.660	0.610	0.500	5	0.685	0.635	0.450
GBPFM201	3	0.421	0.365	0.725	3	0.615	0.534	0.450	3	0.405	0.368	0.750
Mean	3.8	0.402	0.368	0.710	4.2	0.583	0.531	0.539	3.7	0.437	0.403	0.690

GD: Genetic diversity, PIC: Polymorphism Information Content, MAF: Major allele frequency.

Analysis of molecular variance (AMOVA) to assess genetic differentiation both between and within the three types, consisting of 40 CF accessions, 20 WF accessions, and 20 WC accessions within the *Perilla* accessions, indicated that 34 % of the genetic variations was due to differences among the population. Meanwhile, the remaining 66 % of the genetic variation was found to be expressed within the population (Table 3).

**Table 3:** Analysis of molecular variance (AMOVA) based on SSR marker among 80 accessions of three types of the *Perilla* collected from South Korea.

Source	df	SS	MS	Est. Var.	%
Among Pops	2	551.163	275.581	10.229	34 %
Within Pops	77	1528.525	19.851	19.851	66 %
Total	79	2079.688		30.080	100 %

Note: df-Degrees of freedom; SS-Sum of squares; MS-Mean of squares; Est. Var.-Estimate of variance; %-Percentage of total variation.

### 3.2 PSA and phylogenetic relationships among the three types (CF, WF, WC) of the Perilla crop originating from South Korea using Perilla SSR markers

**Fig. 1:**
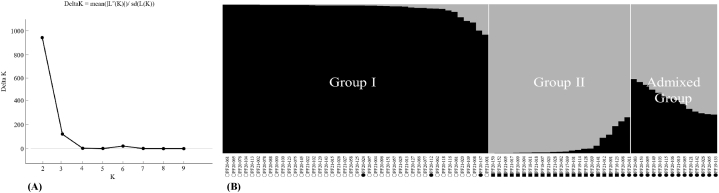
Magnitude of *ΔK* as a function of *K*; the peak value of *ΔK* was at *K* = 2 (A). Population structure of 80 *Perilla* accessions collected from South Korea based on 50 *Perilla* SSRs for *K* = 2 (B). ○: accessions of CF, ●: accessions of WF, ■: accessions of WC.

In our research, we employed a set of 50 *Perilla* SSR loci to examine both the phylogenetic relationships and the PSA among 80 *Perilla* accessions of the three types (CF, WF and WC) originating from South Korea. Within these three types, the STRUCTURE software was utilized, employing ΔK as the guiding criterion for optimal subgrouping and effectively classifying each *Perilla* accession into its respective subgroup. The highest *ΔK* value, indicating the most optimal number of groups, was found to be *K* = 2 for all 80 Korean *Perilla* accessions (Fig. 1A). When a membership probability threshold of 0.75 was set, the 80 *Perilla* accessions of the three types (CF, WF, WC) were classified into two main groups (Groups I, II), along with an admixed group, at *K* = 2 (Fig. 1B). Group I included 43 accessions, consisting of 40 CF accessions and 3 WF accessions. Group II comprised 23 accessions, including 20 WC accessions and 3 WF accessions, and the admixed group included 14 WF accessions (Fig. 1B).

**Fig. 2:**
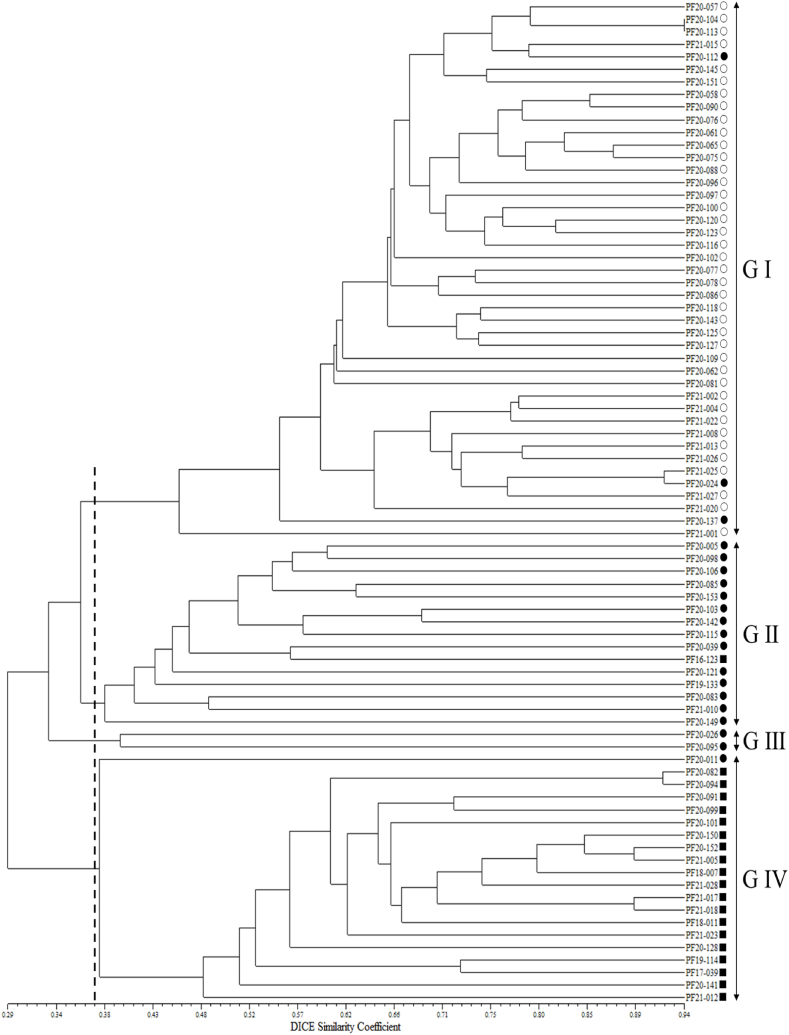
UPGMA dendrogram of 80 accessions of three types (CF, WF, WC) of the *Perilla*collected from South Korea based on 50 *Perilla* primer sets. ○: accessions of CF, ●: accessions of WF, ■: accessions of WC.

In the analysis of a phylogenetic tree constructed using the UPGMA algorithm, the 80 *Perilla* accessions belonging to the three types (CF, WF, WC) were classified into four major clusters, representing a GS value of 37.2 % (Fig. 2). Group I comprised 40 CF accessions and three WF accessions. Group II consisted of 15 accessions, comprising 14 WF accessions and one WC accession. Group Ⅲ comprised two WF accessions. Group IV included 20 accessions, composed of 19 WC accessions and one WF accession (Fig. 2).

### 3.3 AMA of SSR markers and leaf aromatic substance characteristics among accessions of the three types (CF, WF, WC) of the Perilla crop collected from South Korea

In a prior study by Sa et al. [5], researchers identified 41 volatile compounds from 10 chemical classes in the 80 *Perilla* accessions of the three types (CF, WF, WC). The accessions of the three types (CF, WF, WC) of the *Perilla* showed respective totals of 34, 39, and 41 of volatile compounds. PK was the most abundant compound in CF (87.2 %) and WF (64.5 %) while PA was the main compound in WC (26.4 %). In addition, the amounts/levels/ratios of 29 substances differed significantly between WC and CF, and the amounts/levels ratios of 27 substances differed between WC and WF. Based on the volatile compounds, CF accessions were classified as the PK type, while WC accessions were identified as either PA or phenylpropanoid (PP) types. WF accessions, displaying both PK and PP chemical composition types, exhibited intermediate characteristics in their volatile compound compositions in comparison with CF and WC (Supplementary data, Table S3).

Furthermore, Sa et al. [5] investigated the characteristics of volatile compounds between the three types (CF, WF, WC) of the *Perilla* accessions. In CF, PK (87.2 %) was identified as the predominant compound. In WF, the predominant compounds were PK (64.5 %), myristicin (7.0 %), and dill apiol (5.3 %). For WC, the leading components were PA (26.4 %), dill apiol (13.8 %), and (Z,E)-α-farnesene (9.2 %) (Supplementary data, Table S3). Therefore, in this study, a correlation analysis was performed on the five components [PA, PK, myristicin, dill apiol, (Z,E)-α-farnesene] that had a relatively high content in the 80 *Perilla* accessions of the three types (CF, WF, WC) collected within South Korea (Table 4).

**Table 4:** Pearson correlation matrix for five volatile compounds in 80 accessions of three types (CF, WF, WC) of the *Perilla*.

	Dill apiol	Myristicin	Perilla ketone	(Z,E)-α-farnesene
Perilla aldehyde	−0.107	−0.084	−0.583^b^	0.262^a^
Dill apiol		0.125	−0.470^b^	0.174
Myristicin			−0.395^b^	0.165
Perilla ketone				−0.584^b^

a Significant at P < 0.05.

b Significant at P < 0.01.

During the analysis, it was observed that among the 80 *Perilla* accessions of the three types (CF, WF, WC), five volatile compounds displayed positive or negative correlation coefficients that were statistically significant, with significance levels set at 0.05 and 0.01. Among the five volatile compounds, the combinations of PA and PK (−0.583**), dill apiol and PK (−0.470**), myristicin and PK (−0.395**), and PK and (Z, E)-α-farnesene (−0.584**) exhibited the highest negative correlation coefficients when compared with the remaining combinations, and all of them were statistically significant at the 0.01 significance level. In contrast, among the various combinations considered, only the combination of PA and (Z,E)-α-farnesene displayed a positive correlation coefficient (0.262*) while the other combinations did not exhibit statistically significant differences at significance levels of 0.01 or 0.05 (Table 4).

Meanwhile, the 50 *Perilla* SSR primer sets and the five volatile compounds [PA, PK, myristicin, dill apiol, (Z,E)-α-farnesene] that had relatively high contents in the three types (CF, WF, WC) of *Perilla* accessions were used to perform AMA (Supplementary data, Table S4). The TASSEL statistical program was utilized to identify significant marker-trait associations (MTAs) resulting from the AMA of the 50 *P*erilla SSR markers and five volatile compounds. The AMA results revealed a total of 126 MTAs, with 32 associated with PA, 40 with PK, 10 with myristicin, 18 with dill apiol, and 26 with (Z,E)-α-farnesene. These associations were initially detected using a Q GLM at levels of significance of P ≤ 0.05 and 0.01, as detailed in Supplementary data, Table S4. To ensure the reliability of our findings and avoid false positives, we also employed the Q + K MLM analysis method at the same significance levels (P ≤ 0.05 and 0.01). The findings revealed 13 *Perilla* SSR markers that exhibited associations with the five volatile compounds (P ≤ 0.05 or P ≤ 0.01) (Table 5). Additionally, we identified significant 11 MTAs overlapping between the Q GLM and Q + K MLM analyses at the same significance levels (P ≤ 0.05 and 0.01). Among these MTAs, five *Perilla* SSR markers (KNUPF11, KNUPF23, KNUPF32, KNUPF146, KNUPF166) were associated with PA, three SSR markers (KNUPF23, KNUPF37, KNUPF42) were associated with dill apiol, and three SSR markers (KNUPF33, KNUPF125, KNUPF168) were related to (Z,E)-α-farnesene (Table 5). Also, KNUPF42 and KNUPF20 were associated with myristicin and PK, respectively, only in the Q + K MLM analysis.

**Table 5:** Information on significant MTA markers for five volatile compounds using the GLM and MLM methods for 80 accessions of the three types of *Perilla*.

Trait	Marker	GLM	MLM
Perilla aldehyde	KNUPF11	**	*
	KNUPF23	**	**
	KNUPF32	**	**
	KNUPF146	**	*
	KNUPF166	*	**
Dill apiol	KNUPF23	**	**
	KNUPF37	**	*
	KNUPF42	*	**
Myristicin	KNUPF42		*
Perilla ketone	KNUPF20		*
(Z,E)-α-farnesene	KNUPF33	*	*
	KNUPF125	**	*
	KNUPF168	**	*

*P ≤ 0.05, **P ≤ 0.01.

## 4 Discussion

### 4.1 Assessment of genetic variation and volatile compounds in the three types of the Perilla crop using Perilla SSR markers and GC-MS

The growing interest in functional foods has led to a greater focus on studying the effects of plant-derived compounds on human health. Recently *Perilla* crop has been found to have several important effects on the body, including anxiolytic, antidepressant, anti-anxiety, anti-inflammatory, antibacterial, antitumor promoting, and chemo-preventive effects [5,12,[37], [38], [39]]. These effects are due to the abundance of bioactive compounds found in *Perilla* plants, including unsaturated fatty acids (ω-3 fatty acids), essential oils (volatile compounds), flavonoids, phenolic compounds, and triterpenes [11,40]. Among these compounds, the volatile compounds contained in *Perilla* crop are especially significant due to their contributions to aroma, flavor, and medicinal properties [5,16,41]. As a result, these volatile compounds are widely utilized in the pharmaceutical and food additive industries [5,42,43].

Recently in South Korea there has been great interest in developing high-quality cultivars for leafy vegetables because of the expansion of use of *Perilla* leaves as fresh leafy vegetables. Research institutes of the Rural Development Administration (RDA) of South Korea are conducting joint research with universities to discover useful breeding materials with leaves of excellent fragrance among the germplasm of the three types of the *Perilla* crop for the development of domestic leafy vegetable varieties. As part of these efforts, many researchers have analyzed directional substances found in various genetic sources of *Perilla* crop. In a recent study carried out by Sa et al. [5], a comprehensive analysis utilizing GC-MS revealed the presence of 41 volatile compounds belonging to 10 distinct chemical classes in the 80 *Perilla* accessions of the three types (CF, WF, WC) collected from South Korea (Supplementary data, Table S3). They reported that all of the 41 compounds were present in 20 WC accessions of *Perilla*, but seven specific compounds (dill apiol, elemicin, myristicin, PA, shisool, α-terpineol, β-pinene) were absent in 20 CF accessions of *Perilla* (Supplementary data, Table S3). Furthermore, when comparing the statistical differences in the concentrations of commonly identified compounds between CF and WC, significant variations were observed in the levels of 29 volatile compounds [5].

Both CF and CC belong to the same *Perilla* species, and the difference in food use between these two types in South Korea and Japan is because of the plant aromas of both CF and CC types. Variations in chemical compounds like flavonoids, unsaturated fatty acids, and essential oils (volatile compounds) significantly contribute to the differentiation in uses and diversity between CF and CC or WC of the *Perilla* crop [5,11,40]. Therefore, in order to facilitate the development of premium leafy vegetable cultivars, it is essential to analyze the characteristics of the volatile compounds and genetic relationships in the germplasm of *Perilla* crop.

In our present investigation, our primary objective was to pinpoint specific *Perilla* SSR markers that exhibit associations with aromatic substances present in *Perilla* leaves collected from South Korea. To achieve this, we examined the genetic makeup, specifically the genotypes, of 50 *Perilla* SSR primer sets along with the content of five volatile compounds [(PA, PK, myristicin, dill apiol, (Z,E)-α-farnesene)] in 80 *Perilla* accessions of the three types (CF, WF, WC) collected from South Korea. *Perilla* SSR primer sets for *Perilla* crop have been published by different researchers [[19], [20], [21], [22],^44^,^45^]. From this pool of previously reported *Perilla* SSR primer sets, as a primary test we selected 50 *Perilla* SSR loci that demonstrated high amplification of DNA fragments and yielded clear banding patterns in *Perilla* accessions.

According to the findings from this study involving 80 *Perilla* accessions of the three types (CF, WF, WC) gathered from South Korea, the 50 *Perilla* SSR primer sets proved to be valuable molecular markers for assessing GD and genetic relationships. These SSR loci revealed a total of 276 alleles, averaging 5.5 alleles per locus across the 80 *Perilla* accessions of the three types (CF, WF, WC) (Table 1). The allele counts per SSR locus observed in this study are comparable to or lower than those reported in previous research on *Perilla* accessions, such as 9.2 alleles [46], 7.9 alleles [47], 5.9 alleles [21], and 5.8 alleles [25]. The reason for this discrepancy is likely to be because the *Perilla* SSR loci used in this study were specifically chosen to produce clear and distinct DNA fragments, based on preliminary experiments conducted with various *Perilla* SSR primer sets that were developed previously.

In addition, average GD values for the *Perilla* accessions of the three types (CF, WF, WC) were 0.402, 0.583, and 0.437, respectively (Table 2). These results show the highest genetic variation in the WF accessions compared with both the CF and WC accessions, which is consistent with previous reports by Lee and Ohnishi [7], Sa et al. [46], Fu et al. [21], and Jang et al. [26]. Namely, the genetic diversity values of the three types (CF, WF, WC) were reported to be 1.07, 2.23, and 1.75 based on the AFLP markers [7], 0.549, 0.685, and 0.557 [46], 0.331, 0.588, and 0.389 [21], and 0.283, 0.559, and 0.393 [26] based on the SSR markers, respectively. Based on these findings, the researchers concluded that the weedy *Perilla* accessions, represented by the WF type, are valuable genetic resources for the development of cultivated varieties in South Korea. Also, the genetic resources of WF are thought to be well preserved in South Korea even now. Therefore, these results will provide useful information for strategies for collecting and conserving *Perilla* genetic resources in South Korea.

### 4.2 Genetic relationships and AMA of leaf aromatic substances among the three types of the Perilla crop using Perilla SSR markers

In our research, we aimed to enhance our comprehension of the evolutionary relationships among three types (CF, WF, WC) of the *Perilla* crop and achieved this by analyzing the genetic relationships and population structure of 80 *Perilla* accessions. When examining the UPGMA dendrogram depicting these accessions (Fig. 2), distinct clustering patterns emerged, clearly classifying the three types (CF, WF, WC), except for several accessions.

In addition, in the PSA of the three types of the *Perilla* crop, the 80 *Perilla* accessions were categorized into two main groups (Groups I, II), along with an admixed group, at *K* = 2 (Fig. 1B) as follows: Group I comprised 43 accessions, comprising 40 CF accessions and 3 WF accessions. Group II consisted of 23 accessions, consisting of 20 WC accessions and 3 WF accessions. The admixed group comprised only 14 WF accessions (Fig. 1B). Therefore, our results show that, with only a few exceptions, most accessions between the three types (CF, WF, WC) of the *Perilla* crop were clearly distinguished based on both PSA (Fig. 1B) and phylogenetic relationships (Fig. 2). The findings presented here are consistent with previous studies performed by Lee et al. [3], Lee and Ohnishi [7], Sa et al. [46], Fu et al. [21], and Jang et al. [26]. They suggested that exceptional accessions might have originated as escapees from cultivation or probably emerged as hybrids resulting from crosses between accessions of the three types (CF, WF, WC) of the *Perilla* crop. In relation to the case of hybrids, it has been reported that the members of the three types have the same number of chromosomes (2n = 40) and can be interbred by artificial crossbreeding between CF and WF or WF and WC [19,48,49].

**Fig. 3:**
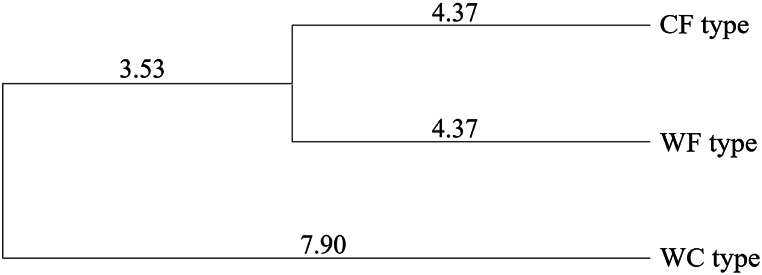
Phylogenetic tree between three types (CF, WF, WC) of the *Perilla* based on Nei's genetic distance.

Furthermore, an interesting result from this study is that although the differentiation process of both cultivated types (CF, CC) and weedy types (WF, WC) of the *Perilla* crop in East Asia remains unclear, the WC type is located at the outermost branch of the phylogenetic tree and the CF and WF types are more closely related to each other than to the WC type, as shown in Fig. 3. In particular, in the analysis of volatile compounds for the three types (CF, WF, WC) of the *Perilla* crop, it was intriguing to observe that WF accessions (displaying PK), in comparison with CF and WC accessions, demonstrated intermediary characteristics in the composition of volatile compounds (Supplementary data, Table S3). These results suggest that the CF and WF types might be derived from the WC type, or the WF type could have arisen from hybrids between the CF and WC types. According to previous reports by Lee and Ohnishi [2], Ha et al. [23], Fu et al. [50], and Jang et al. [26], the WF and WC (or CC) types have smaller seed size and stronger seed dormancy than the CF type, whereas the CF type has large seed size and almost no dormancy. Therefore, these weedy types (WF and WC) are considered likely to play a pivotal role in elucidating the origins of the *Perilla* crop, as noted in previous reports by Lee and Ohnishi [7], Lee et al. [3], Sa et al. [20], and Ha et al. [23]. In conclusion, our findings indicate that genetic variation in *Perilla* species is mainly influenced by the gene exchange processes or gene flow occurring between the three types (CF, WF, WC) of the *Perilla* crop collected from South Korea.

Finally, in our research, we conducted a study to identify specific DNA markers (*Perilla* SSR markers) associated with aromatic substances found in *Perilla* leaves. We examined 80 different accessions of *Perilla* plants belonging to three types (CF, WF, WC) of the *Perilla* crop collected from South Korea. The aromatic substances we focused on were five volatile compounds: PA, PK, myristicin, dill apiol, and (Z,E)-α-farnesene. According to the AMA results between 50 *Perilla* SSR primer sets and the five volatile compounds, we detected a total of 126 MTAs, including 32 for PA, 40 for PK, 10 for myristicin, 18 for dill apiol, and 26 for (Z,E)-α-farnesene using a Q GLM with a level of significance of P ≤ 0.05 and 0.01 (Supplementary data, Table S4).

However, to ensure the accuracy of our findings and avoid false results, alongside the Q GLM analysis method we also employed a Q + K MLM analysis method. From the outcomes presented in Table 5, we pinpointed 13 *Perilla* SSR markers linked to the five volatile compounds (P ≤ 0.05 or P ≤ 0.01). Additionally, we found that 11 of these significant MTAs were common between the Q GLM and Q + K MLM analyses. However, two *Perilla* SSR markers, KNUPF42 and KNUPF20, were only associated with myristicin and PK, respectively, in only the Q + K MLM analysis. Among the *Perilla* SSR markers that were linked to the aromatic substances in *Perilla* leaves, one marker (KNUPF23) was linked to both PA and dill apiol traits, while another marker (KNUPF42) was linked to both dill apiol and myristicin traits (Table 5). In addition, among the 13 *Perilla* SSR markers linked to the five volatile compounds, five markers (KNUPF11, KNUPF23, KNUPF32, KNUPF146, KNUPF166) were linked to PA, three markers (KNUPF23, KNUPF37, KNUPF42) were linked to dill apiol, one marker (KNUPF42) was linked to myristicin, one marker (KNUPF20) was linked to PA, and three markers (KNUPF33, KNUPF125, KNUPF168) were linked to (Z,E)-α-farnesene.

Furthermore, we observed that certain combinations of the five volatile compounds showed high negative correlation coefficients. For example, as shown in Table 4, the combinations between PA and PK (−0.583**), dill apiol and PK (−0.470**), myristicin and PK (−0.395**), and (Z,E)-α-farnesene and PK (−0.584**) exhibited the highest negative correlation coefficients compared with the other combinations, at significance levels of 0.01. Meanwhile, based on the analysis using the Q GLM method, among the 126 MTAs [32 for PA, 40 for PK, 10 for myristicin, 18 for dill apiol, 26 for (Z,E)-α-farnesene], most *Perilla* SSR markers were linked together in two or more aromatic substance traits as follows: 32 SSR markers linked to PA, 18 SSR markers linked to dill apiol, 10 of the 11 SSR markers linked to myristicin, and 25 SSR markers linked to (Z,E)-α-farnesene were also associated with the PK trait (Supplementary data, Table S4). This finding suggests that these *Perilla* SSR markers could be valuable molecular tools for distinguishing the five volatile compounds in *Perilla* plants from the three types (CF, WF, WC) of the *Perilla* crop collected in South Korea.

Although the amount of SSR primer sets developed for *Perilla* crop is relatively small compared with those for other important crops, the SSR primer sets used in this study provided essential information for creating DNA markers linked to five volatile compounds and for understanding genetic variations (or characteristics) among the three types (CF, WF, WC) of the *Perilla* crop grown in South Korea. Therefore, this current study revealed that specific genetic markers (*Perilla* SSR markers) were consistently associated with multiple aromatic compounds in *Perilla* leaves. These genetic markers could be helpful for identifying and differentiating between the five volatile compounds in *Perilla* plants and for understanding the genetic relationships among the three types (CF, WF, WC) of the *Perilla* accessions collected from various regions in South Korea. Moreover, these *Perilla* SSR markers developed in this research may be valuable for identifying key genes/QTLs in *Perilla* crop breeding programs, and enable breeders to enhance leaf and aroma quality using marker-assisted selection breeding methods.

## 5 Conclusion

In the *Perilla frutescens* species, the two varieties are clearly distinguished by their aromatic substances and have different uses in East Asia. An association analysis using SSR markers was performed on volatile compounds in *Perilla* leaves that show differences between CF, WF, and WC of *Perilla*, and it revealed differences among the three types (CF, WF, WC) of the *Perilla* accessions. In South Korea, WF accessions exhibited the greatest genetic diversity among the three types (CF, WF, WC) of the *Perilla* accessions. Phylogenetic tree analysis further divided the 80 *Perilla* accessions into four distinct groups with a genetic similarity of 37.2 %. Three types (CF, WF, WC) of the *Perilla* crop were distinctly categorized except for outstanding accessions. By conducting an AMA of the 50 SSR markers and five volatile compounds (perilla aldehyde, perilla ketone, myristicin, dill apiol, (Z,E)-α-farnesene) in the three types of the *Perilla* crop, we detected 11 significant marker-trait associations duplicated in both Q GLM and Q + K MLM methods. The *Perilla* SSR primers employed in this research furnished valuable insights into developing DNA markers linked to the five volatile compounds and genetic variation (or characteristics) among the 80 *Perilla* accession of the three types (CF, WF, WC) collected from South Korea. These findings serve as valuable insights for identifying the aromatic substances in *Perilla* plants originating from various regions of South Korea.

## Additional information

No additional data and information is available for this paper.

## Funding

This study was supported by a 10.13039/501100003725National Research Foundation of Korea (10.13039/501100003725NRF) grant funded by the Korean government (10.13039/501100014188MSIT) (No. 2022R1F1A1063300) and the Cooperative Research Program for Agriculture Science & 10.13039/100006180Technology Development (project no. PJ01422701), Rural Development Administration, Republic of Korea.

## Data availability statement

The authors declare the data is included in the article and no additional data is available.

## CRediT authorship contribution statement

**Jungeun Cho:** Writing – original draft, Conceptualization. **Kyu Jin Sa:** Writing – original draft, Conceptualization. **Hyeon Park:** Conceptualization. **Tae Hyeon Heo:** Conceptualization. **Sookyeong Lee:** Resources, Data curation. **Ju Kyong Lee:** Writing – review & editing, Writing – original draft, Investigation.

## Declaration of competing interest

We declare that this manuscript is original, has not been published and is not under consideration for publication elsewhere and there are no conflicts of interest to disclose.
